# Ligand-modified homologous targeted cancer cell membrane biomimetic nanostructured lipid carriers for glioma therapy

**DOI:** 10.1080/10717544.2021.1992038

**Published:** 2021-10-20

**Authors:** Mengyu Chen, Yuexin Cui, Wenyan Hao, Yueyue Fan, Jingqiu Zhang, Qianqian Liu, Mingrui Jiang, Yang Yang, Yingzi Wang, Chunsheng Gao

**Affiliations:** a School of Chinese Materia Medica, Beijing University of Chinese Medicine, Beijing, People’s Republic of China; b State Key Laboratory of Toxicology and Medical Countermeasures, Beijing Institute of Pharmacology and Toxicology, Beijing, People’s Republic of China

**Keywords:** Nanostructured lipid carriers, cancer cell membranes, biomimetic drug-delivery system, gliomas, blood–brain barrier

## Abstract

The main treatment measure currently used for glioma treatment is chemotherapy; the biological barrier of solid tumors hinders the deep penetration of nanomedicines and limits anticancer therapy. Furthermore, the poor solubility of many chemotherapeutic drugs limits the efficacy of antitumor drugs. Therefore, improving the solubility of chemotherapeutic agents and drug delivery to tumor tissues through the blood–brain barrier (BBB) and blood–brain tumor barrier (BBTB) are major challenges in glioma treatment. Nanostructured lipid carriers (NLCs) have high drug loading capacity, high stability, and high *in vivo* safety; moreover, they can effectively improve the solubility of insoluble drugs. Therefore, in this study, we used solvent volatilization and ultrasonic melting methods to prepare dihydroartemisinin nanostructured lipid carrier (DHA-NLC). We further used the glioma C6 cancer cell (CC) membrane to encapsulate DHA-NLC owing to the homologous targeting mechanism of the CC membrane; however, the targeting ability of the CC membrane was weak. We accordingly used targeting ligands for modification, and developed a bionanostructured lipid carrier with BBB and BBTB penetration and tumor targeting abilities. The results showed that DHA-loaded NGR/CCNLC (asparagine–glycine–arginine, NGR) was highly targeted, could penetrate the BBB and BBTB, and showed good anti-tumor effects both *in vitro* and *in vivo*, which could effectively prolong the survival time of tumor-bearing mice. Thus, the use of DHA-loaded NGR/CCNLC is an effective strategy for glioma treatment and has the potential to treat glioma.

## 1. Introduction

Cancer is a major threat to human health worldwide. Among the several types of cancers, gliomas are the most common intracranial malignant tumors, with the characteristics of high recurrence rate, high mortality rate, and limited treatment options (Cheng et al., 2020; Jaraiz-Rodriguez et al., 2020; De Witt Hamer et al., 2021; Ganganboina et al., 2021). Chemotherapy has become one of the most important treatment methods for cancer given its high efficacy (Gu et al., 2014; Tan et al., 2020). In recent years, natural drugs and their related active ingredients have been gradually discovered as potential sources of chemotherapeutic agents affecting a variety of tumors (Bishayee & Sethi, 2016; Xiao et al., 2016).

Dihydroartemisinin (DHA) is an active metabolite of artemisinin, with a high efficacy and low toxicity (Chen et al., 2019), and can exert anti-tumor effects by regulating the cell cycle, inducing apoptosis, and inhibiting angiogenesis and other pathways (Liu et al., 2015a; Wan et al., 2019). It has a significant cytotoxic effect on glioma cells with little effect on normal cell metabolism. Further, it has no cross-resistance to traditional therapeutic drugs, and can reverse the multi-drug resistance of tumor cells (Que et al., 2017). However, DHA has disadvantages such as poor solubility and a short circulation time after treatment (Kumar et al., 2019; Li et al., 2020). Moreover, the concentration of drugs reaching the glioma tumors is limited by the blood–brain barrier (BBB) and blood–brain tumor barrier (BBTB), making it difficult to reach effective therapeutic concentrations and further increasing the challenge of glioma treatment (Gao, 2016, 2017; Huo et al., 2020; Lu et al., 2020; Ruan et al., 2021). Therefore, solving the problem of DHA solubility, facilitating the transport through the BBB and BBTB, and accumulating the drug at the tumor site are key factors in the treatment of glioma.

Nanomedicine-based tumor-targeted therapy can address the problem of insufficient specificity of chemotherapeutic drugs (Shi et al., 2011; Layek et al., 2016). Nanostructured lipid carriers (NLCs) are used as a new generation of lipid nanoparticles with low *in vivo* toxicity. These are high-quality carriers for insoluble drug delivery and controlled release (Muller et al., 2007; Deng et al., 2020). However, in cancer therapy, NLCs usually enter into the body via the nonspecific targeting of the enhanced permeability and retention (EPR) effect, so that NLCs aggregate at the tumor site with low targeting efficacy. Moreover, NLCs are prone to adsorbing proteins on their surface to form ‘protein crowns’ during blood circulation. These crowns are easily detected as foreign by the innate immune system and then cleared by the reticuloendothelial and mononuclear phagocyte systems (Zhao et al., 2011). Thus, it is difficult for these to cross the BBB and reach the tumor site.

Bionanomaterials, which have good biocompatibility, low toxic side effects, and good biodegradability, have been widely used in various drug delivery systems in recent years, and can effectively avoid the scavenging effect of the body's immune system (Zhang et al., 2013; Que et al., 2019; Wu et al., 2019). Therefore, using new bionanomaterials to wrap the NLCs and construct a bionanomaterial drug delivery system has been proposed (Chen et al., 2016; Jin et al., 2019). Biofilms are used to wrap nanomaterials, and the obtained bionanomaterials retain the biofilm’s integrity for the original membrane proteins, polysaccharides, and lipids so that the bionanomaterials acquire the inherent properties of the biofilms used for wrapping (Sun et al., 2020; Zhao et al., 2020). Among various biofilms, cancer cell (CCs) membranes rely on surface antigens with structural domains that adhere to homologous cells and homologous binding proteins and can overcome environments such as *in vivo* immune clearance and nonspecific adhesion, thus providing immune escape and homologous adhesion capabilities. Moreover, the excellent biocompatibility of the lipids contained in NLCs can increase the adhesion between nanoparticles and cell membranes, resulting in a tighter bond between NLCs and cell membranes, reducing drug leakage, and increasing the drug concentration in tumor tissues and, consequently, the duration of drug action with glioma cells (Ding et al., 2020). Based on this, wrapping CC membranes on the surface of nanomaterials can give nanomaterials immune escape and homologous recognition targeting ability, prolonging the *in vivo* blood circulation time, improving BBB permeability, increasing drug accumulation and retention at tumor sites, and achieving tumor-targeted therapy.

As the CC membrane itself has weak targeting ability, NLCs need to be further modified to improve BBB and BBTB permeability and tumor targeting. Asn-Gly-Arg (NGR) binds specifically to aminopeptidase N (CD13) on endothelial cells during neovascularization. CD13, as a marker of the neovascular system, has a low expression in normal vascular endothelial cells, but is highly expressed in neovascular endothelial cells and some tumor cells (Pierluigi et al., 2018; Valentinis et al., 2019). Therefore, the NGR peptide can target drugs to tumors or angiogenesis tissues, helping the drugs penetrate the BBTB and penetrate and accumulate into tumors.

This study was designed to construct a bionic drug delivery system using NLCs as carriers with receptor-mediated drug delivery and encapsulating drug-loaded NLCs with the C6 CC membrane via active targeting ligand modification for developing anticancer drugs. The insoluble drug DHA was used as a model drug. NLCs were prepared by solvent volatilization and the ultrasonic melting method. Subsequently, C6 cell membranes were coated on dihydroartemisinin nanostructured lipid carrier (DHA-NLC) to obtain DHA-CCNLC, followed by NGR peptide on the cell membrane surface by the lipid insertion method (Seidi et al., 2018). In a mouse model of *in situ* C6 glioma induced by administration via a tail vein injection, the NGR peptide-modified DHA-CCNLC could effectively penetrate the BBB and BBTB, and drug accumulation at the tumor site was found to be significantly higher, providing an effective platform for the targeted delivery of DHA-NLC to glioma.

## 2. Materials and methods

### 2.1. Materials

DHA with greater than 98% purity was provided by Shanghai Yuanye Biotechnology Co., Ltd. (Shanghai, China). Soybean lecithin was provided by Shanghai Yuanye Biotechnology Co., Ltd. (Shanghai, China). DSPE-PEG_2000_-NHS was purchased from Xi’an Ruixi Biotechnology Co., Ltd. (Xi’an, China). Anti-CD44 and anti-CD47 antibodies were purchased from Abcam (Cambridge, UK). Cholesterol was purchased from China Pharmaceutical Group Chemicals Co., Ltd. (Taipei City, Taiwan). Glycerol trioleate and cholesteryl oleate were purchased from Tokyo Chemicals Industry Corporation (Tokyo, China). All chemical reagents were analytical grade.

### 2.2. Cells and experimental animals

Mouse C6 glioma cells (C6), mouse brain microvascular endothelial cells (bEnd.3 cells), human umbilical vein endothelial cells (HUVECs), hepatocellular carcinoma (HepG2) cells, and melanoma cells (B16) were provided by the Cell Resource Centre of IBMS (Beijing, China) and cultured in Dulbecco’s modified Eagle medium (DMEM) containing 10% fetal bovine serum (FBS) and 100 IU penicillin.

Equal numbers of male and female ICR mice weighing 18–22 g were purchased from the SPF Biotechnology Limited Company (License number: SCXK 2019-0010; Beijing, China). All animal experiments were performed in strict accordance with the Regulations for the Management of Laboratory Animals of the Ministry of Science and Technology of the People's Republic of China. The protocol was approved by the Committee on the Ethics of Animal Experiments of the Medical Laboratory Animal Center of the School of Basic Medical Sciences, Beijing University of Traditional Chinese Medicine (No. BUCM-4-2021092204-3092).

### 2.3. Preparation of NLCs

DHA-NLC was prepared by the solvent volatilization ultrasonic melting method. Further, 2.5 mg of DHA, 50 mg of lecithin, 5 mg of triglyceride, 2.5 mg of cholesterol, and 2.5 mg of cholesteryl ester were weighed and dissolved in 5 mL of anhydrous ethanol and 5 mL of acetone as the organic phase; 50 mL of Tris (containing 0.2 M KC1, 2 mM of EDTA, and 0.02 M of Tris–HCl) at pH 8.0 was used as the aqueous phase. The aqueous phase was heated in a water bath at 60 °C and the organic phase was slowly and uniformly added to the aqueous phase under magnetic stirring for 1.5 h (2500 W). The nanoemulsion was then sonicated in an ice-water bath for 5 min (300 W). The sonicated nanoemulsion was concentrated in a water bath at 55 °C under reduced pressure to remove the organic solvent and concentrate the remaining solvent to 10 mL. Finally, the nanoemulsion was allowed to come to room temperature and passed through a 0.45-μm filter membrane to obtain DHA-NLC. NLC loaded with Coumarin 6 (COU6) was prepared in the same manner by replacing DHA with COU6.

### 2.4. Drug loading and encapsulation efficiency

In this study, ultrafiltration centrifugation was used to determine the encapsulation efficiency (EE) and drug loading (DL) of DHA-NLCs. For this, 5 mL DHA-NLC sample was added into the ultrafiltration centrifuge tube, and centrifuged at 5000 r/min for 10 min. The ultrafiltrate was collected and the free drug content (*W*_free_) was determined by high-performance liquid chromatography (HPLC). The same batch of DHA-NLCs was demulsification using methanol, filtered through a 0.22-μm filter membrane, and the total drug content (*W*_total_) was determined under the same chromatographic conditions. EE and DL of DHA-NLC were calculated according to the following formula:

$$\text{EE}\;(\%)=(W_{\text{total}}-W_{\text{free}})/W_{\text{total}}\times 100\%,\;\text{and DL}\;(\%)=(W_{\text{total}}-W_{\text{free}})/(W_{\text{total}}-W_{\text{free}}+W_{\text{lipid}})\times 100\%,$$



where *W*_total_, *W*_free_, and *W*_lipid_ were the weight of total drug in the system, weight of free drug in the filtrate, and weight of lipid added in the system, respectively.

### 2.5. Targeting ligand synthesis

The targeting ligands were synthesized by the active ester method. Using N-hydroxythiosuccinimide (NHS) as the protecting agent, the ester bond in DSPE-PEG_2000_-NHS reacted with the amino group in the NGR peptide under catalyzation by N,N′-dicyclohexylcarbodiimide (DCC) to form an amide bond, and the targeting ligands were lyophilized after dialysis. The synthesis of the targeting ligands was confirmed by ^1^H NMR and MALDI-TOF mass spectrometry (MALDI-TOF MS).

### 2.6. Collection of cancer cell membranes

C6 cells in the logarithmic growth phase were digested with trypsin and centrifuged to extract the precipitate, and the precipitate was collected after washing with phosphate-buffered saline (PBS). A sufficient amount of 25% PBS hypotonic solution containing a protein inhibitor enzyme was added to the collected precipitate, and the CC membranes were obtained by differential centrifugation to remove nuclei and other organelles (Suski et al., 2014).

### 2.7. Preparation and modification of NLCs encapsulated by cancer cell membranes

The prepared CC membranes were centrifuged at 12,000 rpm for 10 min. Following this, the precipitate was collected, resuspended with 5 mL NLC, and fused by ultrasonication (300 W, 5 min, switched on for 2 s and stopped for 5 s) under ice bath conditions to obtain DHA-CCNLC. DHA-NGR/CCNLC was obtained by mixing 2 mg of NGR ligand with NLC wrapped around the CC membranes and incubating for 30 min at 37 °C. DiI and DiR-labeled DHA-CCNLC and DHA-NGR/CCNLC were obtained by adding free DiI and DiR, respectively, to NLC and stirring in dark for 20 min. Free DiI and DiR were removed by 24-h incubation in dark.

### 2.8. Characterization of biomimetic NLCs

The prepared DHA-NLC, DHA-CCNLC, and DHA-NGR/CCNLC were measured separately by dynamic light scattering (DLS) (Litesizer™ 500, Anton-Paar, Graz, Austria) to determine the particle size and zeta potential of each bio-nanocarrier. The NLCs were stored at 4 °C for 30 days to determine their stability. To observe the changes in the crystal structure of the drugs, DHA, blank NLC, DHA-NLC, and the physical mixture of DHA and excipients were lyophilized and detected by X-ray diffraction (XRD, D8 advance, Bruker, Karlsruhe, Germany). The diffraction angle (2*θ*) was 5°–90° and the step was 2°/s. Meanwhile, the existing status of DHA in NLCs was further studied by differential scanning calorimetry (DSC204 HP, Netzsch, Selb, Germany). Subsequently, Fourier transform infrared (FTIR, Spectrum Two, PerkinElmer, Waltham, MA) was used to analyze and identify the drug to determine whether the drug molecule has changed (wavenumber is 4000–450 cm^−1^).

To observe the microscopic morphology of DHA, a small amount of DHA was placed on a double-sided conductive tape, fixed on a short aluminum column, coated with gold, and observed via scanning electron microscopy (SEM, S4800, Hitachi, Shiga, Japan). Further, a small amount of DHA-NLC and DHA-NGR/CCNLC were diluted and dropped onto a copper mesh (300 mesh). After the solvent was dried, 1% phosphotungstic acid was added and dyed for 1 min. The morphology was observed by transmission electron microscopy (TEM, H-7650, Hitachi, Shiga, Japan).

To verify the integrity of the cell membranes on the surface of the biomimetic NLCs, 2 mL each of C6 cell membranes, DHA-NLC, DHA-CCNLC, and DHA-NGR/CCNLC were centrifuged (12,000 rpm, 10 min). The precipitate was collected and a protein lysate was added to the precipitate. The membrane protein content was determined by the BAC method, and the expression of membrane proteins was analyzed by sodium dodecyl sulfate polyacrylamide gel electrophoresis (SDS-PAGE).

CD47 is overexpressed on the surface of CCs and is responsible for immune tolerance and preventing macrophages from clearing CCs, thus helping to shield CCs from the innate immune system and inducing immunosuppression (Pan et al., 2019; Hayat et al., 2020). CD44 is a cell surface glycoprotein involved in intercellular interactions, cell adhesion, and migration, and is associated with tumorigenicity, invasiveness, and lymphatic metastasis of tumor cells (Liu et al., 2015b; Roedig et al., 2020). Both of these are CC surface-specific proteins, which are markers of the functional integrity of the cell membrane. Therefore, we analyzed the contents of CD44 and CD47 by Western blotting to verify the integrity of the cell surface membrane protein function.

### 2.9. *In vitro* release

The *in vitro* release behavior of active pharmaceutical ingredients and formulations was investigated by the dialysis bag method (Yu et al., 2019). The prepared DHA, DHA-NLC, DHA-CCNLC, and DHA-NGR/CCNLC (5 mL each) were placed in a dialysis bag (molecular weight cutoff: 2.5 kDa); then the ends of the bag were sealed and the bag was placed in a 50-mL stoppered centrifuge tube. Buffer solutions at pH 6.8 and pH 7.4 containing 0.5% Tween-80 were selected as the release media to simulate the fluid environment in the body. The shaker was preheated to 37 °C. The samples were placed in the shaker and shaken at 100 r/min. Following this, 2 mL of the buffer solutions were drawn at 5, 15, and 30 min and at 1, 2, 4, 8, 12, and 24 h. Subsequently, the same amount of preheated release medium was immediately added, and samples were taken every 12 h after 24 h until seven days of release. The samples were filtered through a 0.22-μm filter membrane, and the content of the drug was determined by HPLC. The cumulative amount of the drug released was calculated, and the *in vitro* release curve was plotted.

### 2.10. Cell uptake

BEnd.3 cells, HUVEC cells, and C6 cells were used to simulate the BBB and BBTB to investigate the uptake of bionanostructured lipid carriers. COU6-NLC, COU6-CCNLC, and COU6-NGR/CCNLC were incubated with each of these three cells. After 15 min, the cells were fixed with 4% paraformaldehyde and stained with Hoechst 33258. Finally, after sealing the laser confocal dishes with 50% glycerin, confocal laser scanning microscopy (CLSM, LSM 880, Zeiss, Oberkochen, Germany) was performed for observation, and flow cytometry (FCM, FACSAria III, BD, Franklin Lakes, NJ) was performed for quantitative analysis.

### 2.11. *In vitro* cytotoxicity assay

To detect the value-added inhibition of tumor cells by the bionanobionic nanoformulations, the cell counting kit-8 (CCK-8) was used to determine cell viability. C6 cells were inoculated into a 96-well plate at a concentration of 5 × 10^3^ cells/well. After overnight culture, different biomimetic nanodrugs containing 1, 2, 5, 10, 20, 50, or 100 μg/mL of DHA were added to each well. The cells were incubated for 48 h. After removing the medium, 20 μL of CCK-8 solution was added to each well and the plate was incubated for 1–2 h. The absorbance value was then detected using a microplate reader (Tecan Spark, Grödig, Austria) at 450 nm to calculate the cell survival rate.

### 2.12. Homologous targeting

Homologous targeting of the nanoparticles was verified by detecting the uptake efficiency of the biomimetic NLCs by different tumor cells. HepG2 cells, B16 cells, and C6 cells were placed in laser confocal dishes. After overnight culture, equal concentrations of COU6-CCNLC were added to the cell culture medium. After 0.5 h of incubation, the uptake of each cell type was observed via CLSM.

### 2.13. *In vitro* transport across the BBB and BBTB

To investigate the ability of biomimetic nanoformulations to penetrate the BBB, an *in vitro* BBB model was constructed using bEnd.3 and C6 cells construct (Andrews et al., 2018). Here, 1 × 10^5^ b.End3 cells were spread on the upper layer of the Transwell system and cultured until 100% confluence. When the transendothelial resistance reached 300 Ω.cm^2^, 2 × 10^3^ C6 cells were inoculated in the lower layer of the Transwell and cultured until apposition. Following this, COU6-NLC, COU6-CCNLC, and COU6-NGR/CCNLC were added to the upper layer of Transwell chambers and cultured in a cell culture incubator for 4 h. The fluorescence intensity of the lower layer was measured using a microplate reader, and the C6 cells were isolated and stained with Hoechst 33258 at room temperature and observed via CLSM.

After the BBB model was established *in vitro*, DHA, DHA-NLC, DHA-CCNLC, and DHA-NGR/CCNLC were added to the upper chamber at the same concentration. After 48 h of culture, the medium was removed and 20 μL of CCK-8 solution was added to each well, followed by incubation at 37 °C for 1–2 h in the dark. The absorbance value was measured at 450 nm using a microplate reader and the proliferation inhibition rate was determined.

The BBTB *in vitro* model was established using HUVEC cells and C6 cells in the same manner to investigate the ability of the preparation to penetrate the BBTB.

### 2.14. *In vivo* glioma targeting ability

An intracerebral injection of 1 × 10^7^ C6 cells was administered into corpus striatum of the mouse brain to establish an orthotopic implantation model of glioma (Zhai et al., 2018). Following this, 150 μL (*n* = 6) of DiR-loaded DHA-CCNLC, DHA-NGR/CCNLC, or free DiR were injected into the glioma model mice via tail vein injections. At 2, 4, 8, 12, 24, and 36 h after injection, the distribution of DiR fluorescence in the mice was analyzed using the IVIS *in vivo* system (IVIS Spectrum, PerkinElmer, Waltham, MA) and Living Image software (Caliper, Alameda, CA) at an excitation wavelength of 748 nm and an emission wavelength of 780 nm. After 36 h of administration, the brain, heart, liver, spleen, lung, and kidney were promptly removed for *in vitro* imaging and analysis.^®^^®^

The mice were randomly divided into three groups and injected with free DiI, DiI-DHA-CCNLC, or DiI-DHA-NGR/CCNLC through the tail vein. The mice were euthanized 4 h after the injection with biomimetic nanomedicines and the brains were resected for dissection. They were fixed with 4% paraformaldehyde for 24 h and the nuclei were stained with 4′,6-diamidino-2-phenylindole (DAPI), and the fluorescence was analyzed with the excitation wavelength of 358 nm and emission wavelength of 461 nm. The distribution of the nano-suspension in the brain tissue was observed via inverted fluorescence microscopy.

### 2.15. Targeting ability of the ligands

To verify the specific binding of the NGR peptide and ligand receptor, the glioma model mice were divided into two groups. One group was injected with 150 μL of 1 mg/mL free NGR peptide 1 h before the experiment, after which both groups were injected with 150 μL of DiR-DHA-NGR/CCNLC simultaneously. Subsequently, the IVIS *in vivo* system was used to analyze the blocking effect of NGR peptide on the tumor vascular receptor.

### 2.16. *In vivo* circulation time

To evaluate the *in vivo* circulation time of bionanodrugs, the mice were injected with free DiD, DiD-DHA-CCNLC, and DiD-DHA-NGR/CCNLC via the tail vein. Following this, 30 μL (*n* = 6) of orbital blood was collected at 0, 5, 15, and 30 min and 1, 2, 4, 8, 24, and 48 h after injection. The collected blood samples were diluted in a 96-well plate with 70 μL PBS, and the fluorescence was measured using an enzyme marker. Free DiD was mixed with erythrocyte membrane suspension and diluted into 2, 4, 10, 20, and 50 μg/mL solutions. The fluorescence values were determined in relation to the DiD concentration.

### 2.17. *In vivo* anti-glioma

The glioma model mice were randomly divided into five groups (*n* = 10). Following this, 150 µL of saline, free DHA, DHA-NLC, DHA-CCNLC, or DHA-NGR/CCNLC was injected to the mice in each group via the tail vein every two days. The bodyweight of the mice was recorded. After 10 days of administration, the mice were euthanized, and the brains were collected and fixed in 4% paraformaldehyde in dark for 24 h. Hematoxylin and eosin (H&E) staining, CD31 immunohistochemical analysis, caspase 3 assay, and terminal deoxynucleotidyl transferase-mediated dUTP nick end labeling (TUNEL) assay were performed to observe and analyze the damage to tumor tissues in different drug groups.

### 2.18. *In vivo* toxicity evaluation

Normal mice with similar body weights were randomly divided into five groups with three mice in each group. The mice in each group were injected with saline, free DHA, DHA-NLC, DHA-CCNLC, or DHA-NGR/CCNLC into the tail vein every two days. After regular administration for 15 days, blood was collected for blood cell and serum biochemical determination, and the mice were euthanized one day after the last administration. The heart, liver, spleen, lung, kidney, and brain were resected for histopathological analysis to detect the potential toxicity of NLCs *in vivo*.

### 2.19. Statistical analysis

The quantitative data are expressed as means ± standard deviation (SD) unless stated otherwise. One-way analysis of variance (ANOVA) was used to determine significant differences between groups, with statistical significance indicated at *p*<.05.

## 3. Results and discussion

### 3.1. Characterization of DHA-NLC

**Figure 1.**
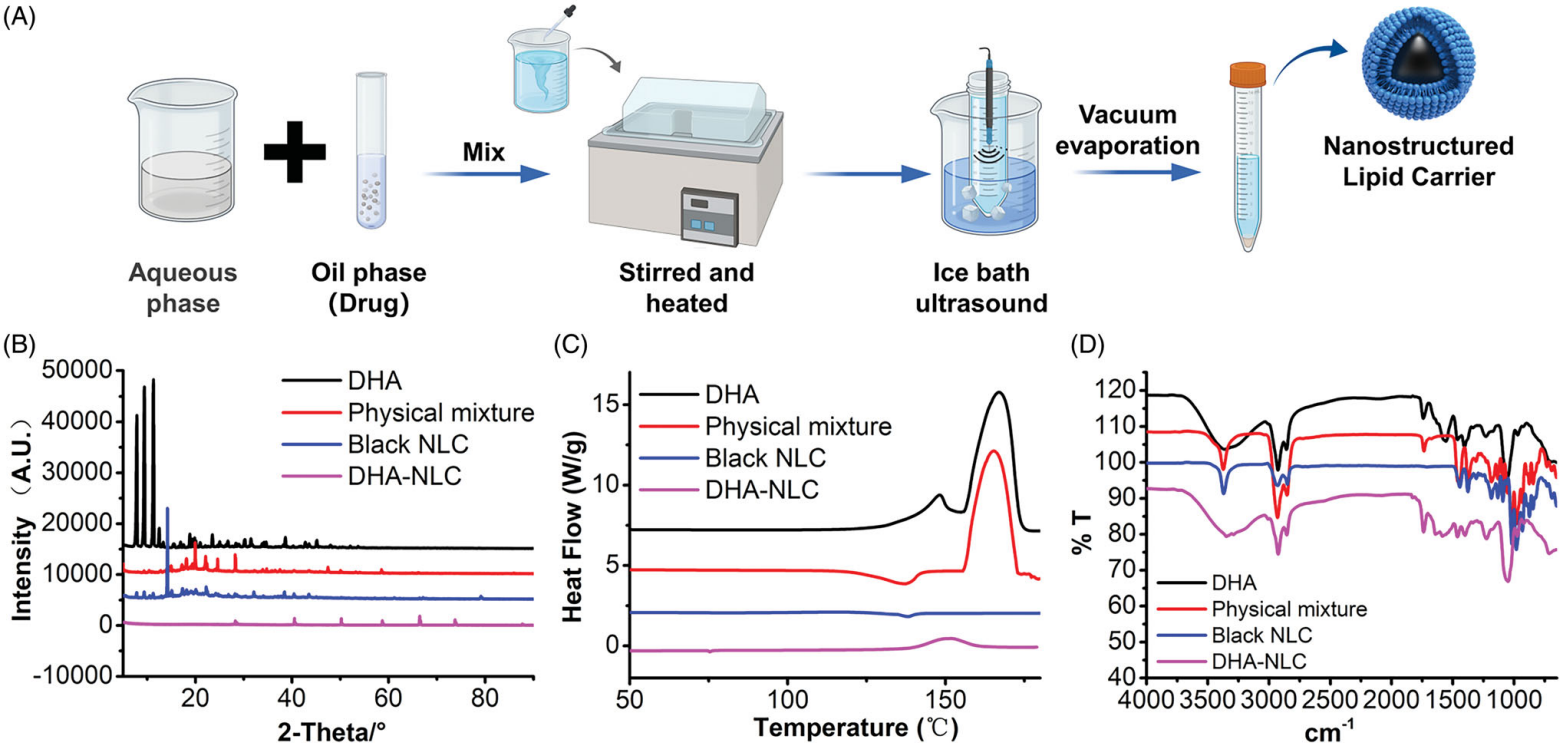
Preparation and characterization of dihydroartemisinin nanostructured lipid carriers. (A) Solid and liquid lipids, surfactants, and drugs were used as the oil phase; stabilizers were dissolved in water; the solvent evaporation ultrasonic melting method was used to prepare nanostructured lipid carriers. X-ray diffraction patterns (B), DSC scanning diagrams (C), and FTIR spectra (D) of different components of the nanostructured lipid carriers.

The preparation of DHA-NLC is illustrated in Figure 1(A). The encapsulation rate of DHA-NLC was determined to be 81.63%, and the DL rate was 3.72%. According to the XRD and DSC results, the original crystal structure of DHA was destroyed after it was prepared as a carrier of nanostructured lipids, and it existed in an amorphous form in NLC (Figure 1(B,C)). The results of infrared spectrum scanning (Figure 1(D)) also proved that DHA only changed its existing form in the preparation process and did not react with the excipients. The results confirmed that DHA was successfully prepared as an NLC.

### 3.2. Characterization of biomimetic NLCs

**Figure 2.**
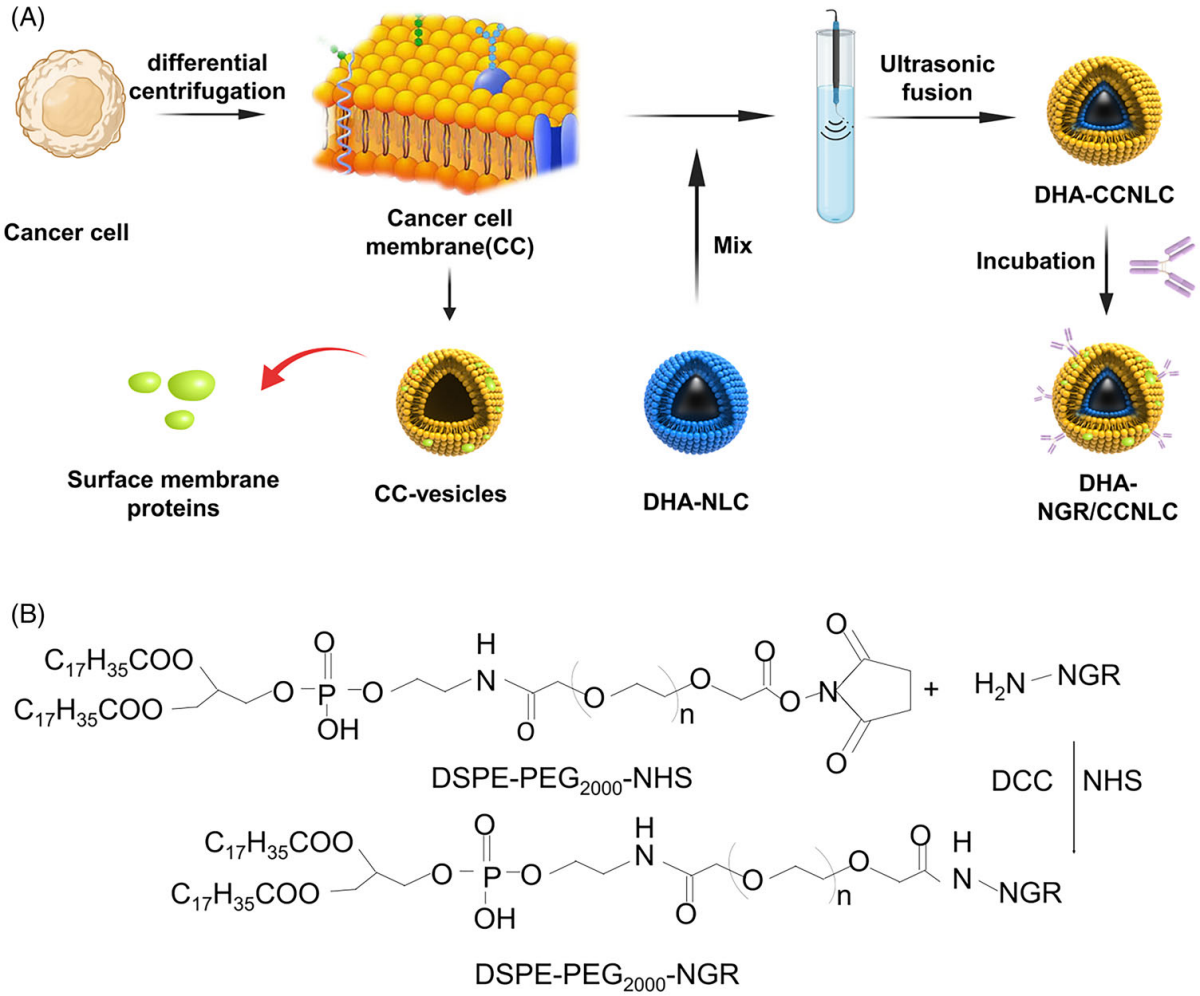
(A) Preparation of DHA biomimetic nanostructured lipid carriers. Cell membranes were obtained based on low permeability and centrifugation and then fused with DHA-NLC combined with ultrasound to obtain a biomimetic nanosuspension. The lipid insertion method was used for active targeting modification. (B) Synthesis of the targeting ligand DSPE-PEG_2000_-NGR. By the action of DCC and NHS, NH_2_ in the polypeptide reacted with the ester in DSPE-PEG_2000_-NHS to form DSPE-PEG_2000_-NGR.

**Figure 3.**
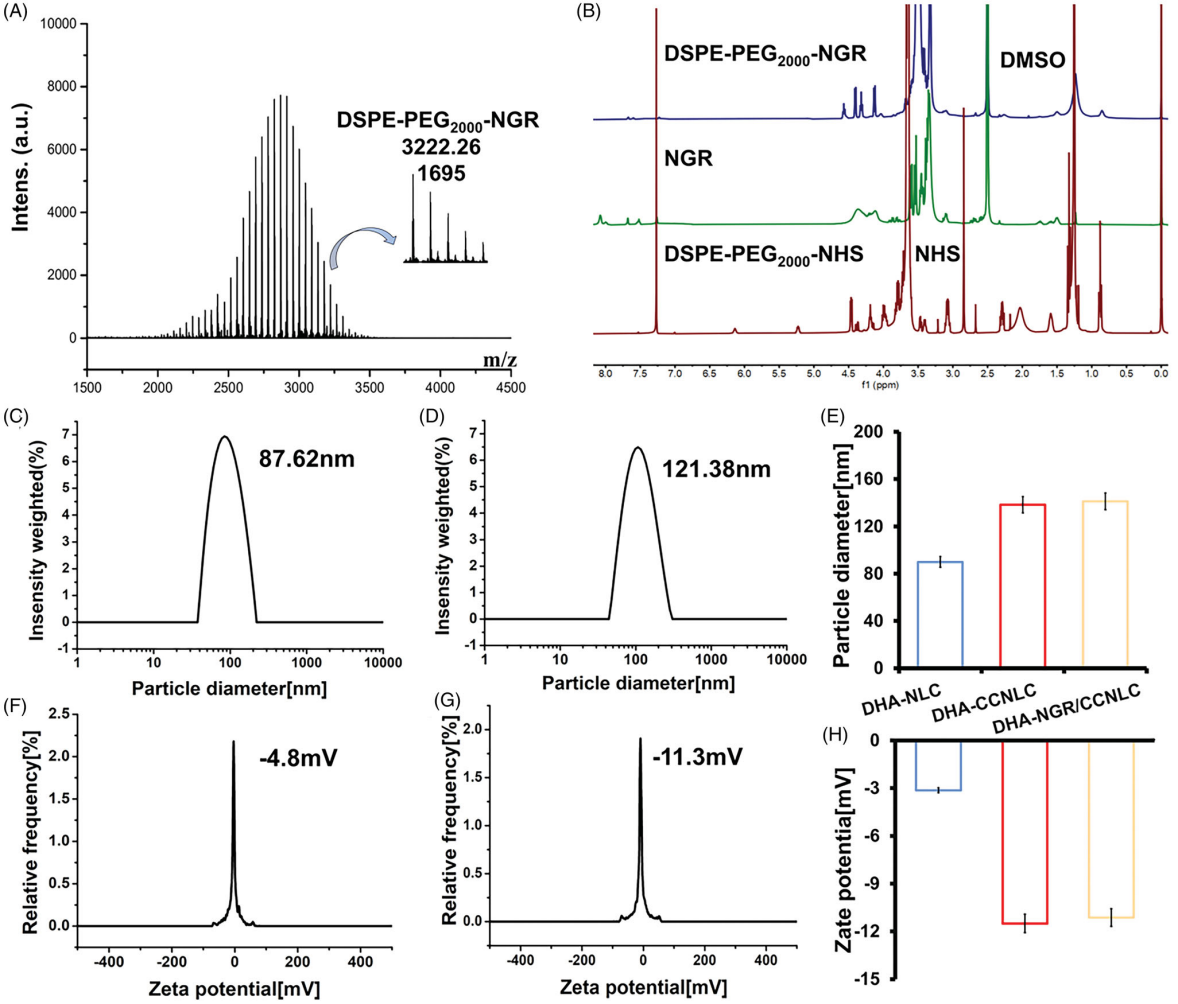
MALDI-TOF MS (A) and ^1^H NMR (B) of DSPE-PEG_2000_-NGR, indicating the successful synthesis of the targeted ligands. The particle size (C) and potential (F) of DHA-NLC. The particle size (D) and potential (G) of DHA-NGR/CCNLC. Diagram of the particle size (E) and potential (H) of the nanostructured lipid carriers before and after modification. The particle size and absolute potential of nanostructured lipid carriers increased after membrane encapsulation.

As shown in Figure 2(A), the CC membrane was fused with the drug nanosuspension via the probe ultrasound technique. The method was simple and reproducible. The synthesis route of DSPE-PEG_2000_-NGR is shown in Figure 2(B). The fluffy powdered targeting ligand was obtained by freeze-drying. Further, successful synthesis of DSPE-PEG_2000_-NGR was confirmed by MALDI-TOF MS. The molecular ion peak with a mass-to-charge ratio of 3222.26 (Figure 3(A)) was observed, which was similar to the molecular weight of DSPE-PEG_2000_-NGR. In ^1^H NMR (Figure 3(B)), the characteristic peak of DSPE-PEG_2000_-NHS disappeared, and the characteristic peak of NGR (*δ* = 3.43) also confirmed the successful synthesis of the target molecule.

After the preparation of the biomimetic NLCs, the particle sizes of DHA-NLC and DHA-CCNLC were approximately 98 nm and 130 nm, respectively. The difference between the two was approximately 30 nm, which was about the thickness of a layer of cell membranes. The difference in potential was approximately 20 mV, which was consistent with the membrane potential, indicating that the surface of the NLCs successfully wrapped the CC membranes. NGR had no obvious effect on the particle size or potential of the biomimetic NLCs (Figure 3(C–H)). The stability results are shown in Figure S1A. It can be seen from the figure that within 30 days, the particle sizes of DHA-NLC, DHA-CCNLC, and DHA-NGR/CCNLC fluctuated within a certain range, and the system was stable.

**Figure 4.**
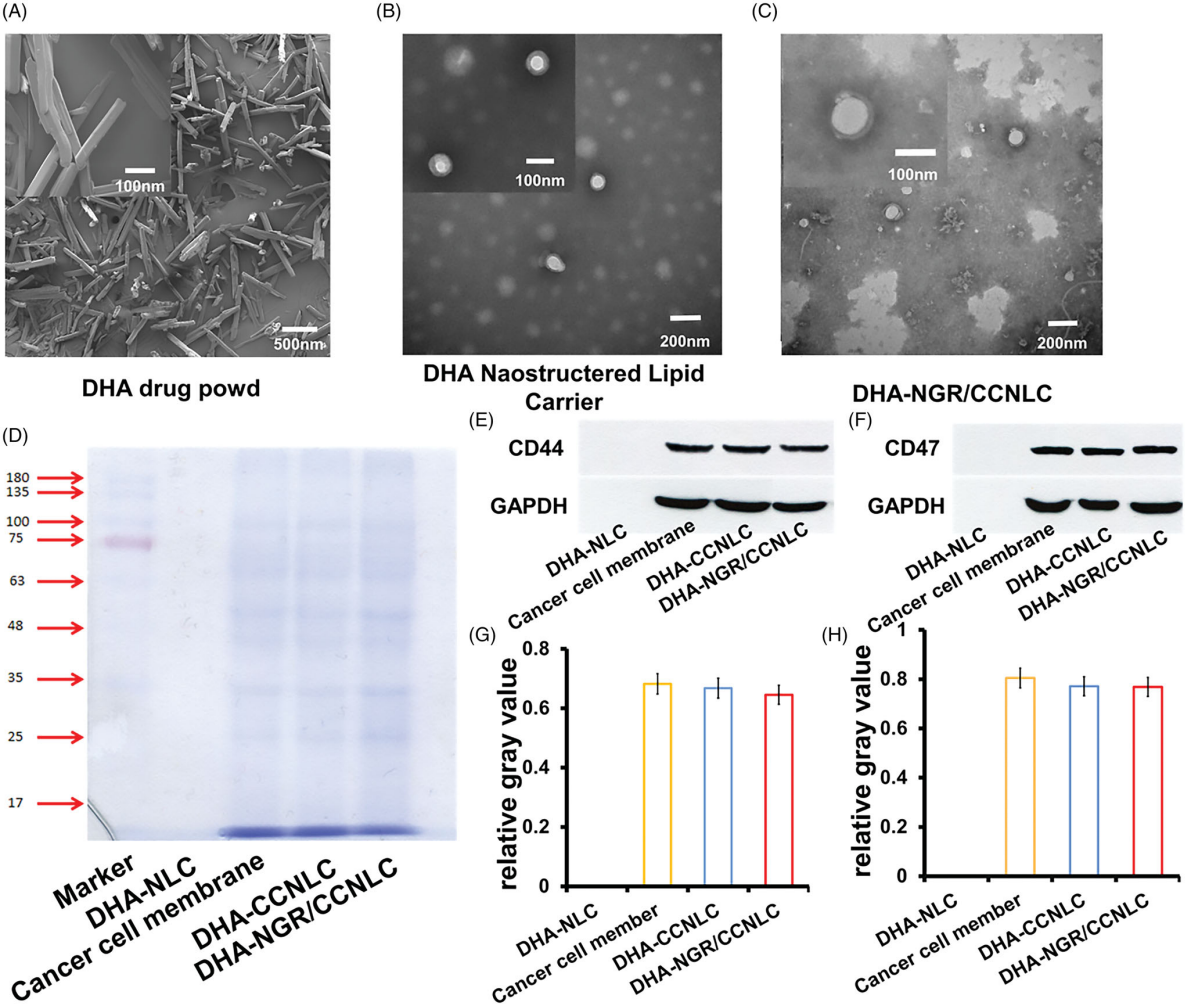
Characterization of biomimetic nanostructured lipid carriers. (A) DHA powder under scanning electron microscopy (SEM) shows a broken prism crystal structure. Transmission electron microscopy (TEM) of DHA-NLC (B) and DHA-NGR/CCNLC (C) shows that DHA-NLC has a core–shell structure, and an obvious membrane structure can be observed after the cancer cell membranes are wrapped. (D) The membrane proteins of the biomimetic nanostructured lipid carriers determined by SDS-PAGE. The Western blotting assay and relative gray values of the membrane-specific CD44 (E, G) and CD47 (F, H) proteins.

DHA showed a crystal structure via SEM (Figure 4(A)). After DHA was prepared into DHA-NLC, an obvious core–shell structure was observed under TEM (Figure 4(B)). The size was uniform, and the crystal structure had disappeared. The particle size was consistent with the results measured using the photodisperse particle size analyzer. There was an obvious membrane structure on the surface of the NLC coated by CCs and modified by the target, forming a stable nanoparticle with a single-layer membrane structure (Figure 4(C)).

The protein component content was analyzed by SDS-PAGE. Figure 4(D) shows that compared with the C6 cell membrane lysates, DHA-CCNLC and DHA-NGR/CCNLC showed consistent bands, with no missing marker protein band. The CC membranes coated on the surface of the NLC were well-retained. CD47 on CC membranes can bind to the corresponding receptors on macrophages to avoid being phagocytized by macrophages (Zhy et al., 2021). Further, CD44 is a specific protein involved in interactions between cells on the surface of CC membranes, and affects the tumorigenesis and metastasis of tumor cells (Thakur et al., 2021). Therefore, determining the existence of and damage caused to CD47 and CD44 is essential for the long-term circulation of the biomimetic nanocarriers *in vivo* and for homologous targeting of CCs. According to the results of CD47 and CD44 assays, DHA-CCNLC and DHA-NGR/CCNLC, but not DHA-NLC, showed the corresponding protein bands. The gray values were analyzed (Figure 4(E–H)). Some NLC protein wrapped by cell membranes was lost, which was consistent with the protein content determined by the BCA method (Figure S1B). It was speculated that some losses were caused during the preparation process. However, no key proteins were lost, and it had no effect on the function of the biomimetic NLCs.

The *in vitro* release results showed that in both release media, the release of free DHA was relatively slow, and the release rate did not reach 50% even after seven days of release. DHA-NLC, DHA-CCNLC, and DHA-NGR/CCNLC showed the release rates that reached more than 70% within 72 h; thus, the release rates of DHA-NLC, DHA-CCNLC, and DHA-NGR/CCNLC are greatly improved compared with the raw material DHA. On comparison between DHA-CCNLC and DHA-NGR/CCNLC groups, no significant difference in the drug release behavior was observed between the target-modified biomimetic nanocarrier group and the unmodified nanocarrier group, indicating that modification with the NGR peptide had no effect on drug release (Figure S1C and D).

### 3.3. Cellular uptake of biomimetic NLCs

**Figure 5.**
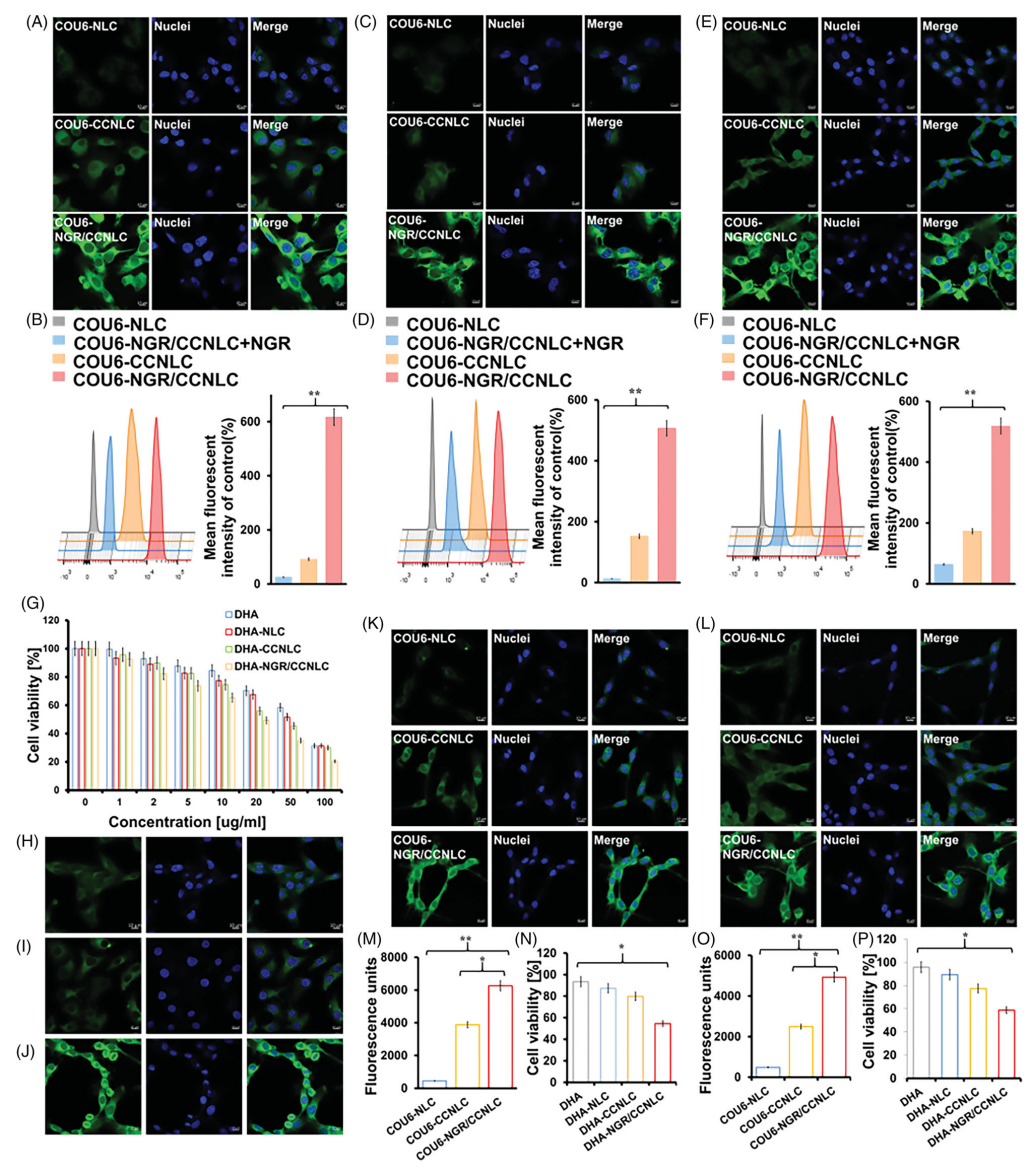
CLSM and FCM were used to investigate cellular uptake. COU6-NLC, COU6-CCNLC, and COU6-NGR/CCNLC were added to bEnd.3 cells (A, B), HUVECs cells (C, D), and C6 cells (E, F), and the tumor-targeting ability of each bionic nanosuspension was determined by CLSM and FCM. *In vitro* BBB and BBTB penetration efficiency. (G) The CCK-8 assay was used to determine the inhibitory effect of drugs on the proliferation of C6 cells, and DHA-NGR/CCNLC showed the best effect (*n* = 5). The uptake of COU6-CCNLC by (H) B16, (I) HepG2, and (J) C6 cells was determined. The *in vitro* BBB model (K) was constructed with bEnd.3/C6 cells and the *in vitro* BBTB model (L) was constructed with HUVECs/C6 cells. The uptake and proliferation inhibition of C6 cells by different drugs were determined by FCM (M, O) and the CCK-8 assay (N, P). CLSM was performed to investigate the uptake of the biomimetic nanosuspension by different cancer cells (×20 objective lens, green: COU6; blue: nuclei; *p<.05, **p<.01).

As shown in Figure 5(A–D), the uptake of COU6-CCNLC and COU6-NGR/CCNLC by HUVEC cells and bEnd.3 cells was significantly stronger than that of COU6-NLC, and the fluorescence intensity of the NGR peptide target-modified biomimetic nanocarriers was the strongest. The free NGR peptide also significantly inhibited the uptake of COU6-NGR/CCNLC by the cells, indicating that the NGR peptide effectively through BBB and targeted tumor, delivered drugs to the brain tumors.

The results of the uptake of the bio-nanocarriers shown in Figure 5(E,F) indicate that compared with the controls, the C6 cells had stronger fluorescence with the uptake of NLC wrapped by CC membranes, showing the homologous targeting ability of tumor cells. The combination of homologous targeting and NGR peptide modification provides active targeting, thus facilitating drug delivery at brain tumor sites.

To investigate the effect of the biomimetic nanopreparations on the growth inhibition of C6 CCs, the CCK-8 assay was performed to investigate the cytotoxicity of raw materials and preparations. It can be seen from Figure 5(G) that the raw drug and nano-preparation have significant concentration dependence, and the inhibitory effect on C6 cells increases with an increase in the drug dose. The IC50 values of DHA, DHA-NLC, DHA-CCNLC, and DHA-NGR/CCNLC were 54.57, 46.09, 34.45, and 20.05 μg/mL, respectively. Among them, the biomimetic nanopreparations modified by the NGR peptide had the strongest inhibitory effect.

### 3.4. Homologous targeting

We further verified the homologous targeting effect of nanoparticles by evaluating the endocytosis effect of nanoparticles in different tumor cells. Figure 5(H–J) shows that HepG2 cells and B16 cells showed less ability to endocytose COU6-CCNLC compared with the C6 cells, which further illustrated that CCNLC had a homologous targeting and specific recognition function, indicating that the selective targeting characteristics of CCNLC were not suitable for other atypical CCs.

### 3.5. *In vitro* BBB and BBTB penetrating ability

*In vitro* BBB and BBTB models were constructed using Transwell chambers to evaluate the ability of the biomimetic nanopreparations to cross the BBB and BBTB. The *in vitro* BBB and BBTB models were determined to have a barrier function with resistance values of 332.7 Ω·cm^2^ and 332.3 Ω·cm^2^, respectively (Srinivasan et al., 2015). As shown in Figure 5(K,L,M,O), the penetration ability of NGR/CCNLC was the strongest and was significantly different compared with that of CCNLC, which proves that the NGR peptide as a targeted ligand is of great significance for penetrating the BBB and BBTB.

Further, the CCK-8 method was used to detect the inhibitory effect of nanoparticles on C6 cells after penetrating the BBB. The results showed that the cell survival rates of DHA, DHA-NLC, DHA-CCNL, and DHA-NGR/CCNLC in the BBB were 93.44%, 87.35%, 79.78%, and 54.59% and those in BBTB were 95.85%, 89.38%, 77.47%, and 58.80%, respectively. The inhibition of C6 cells by DHA-NLC, DHA-CCNLC, and DHA-NGR/CCNLC increased in that order, and the inhibitory effect of the NGR peptide as a target ligand-modified biomimetic nanopreparation on C6 cells was the highest after penetrating the BBB and BBTB (Figure 5(N,P)).

### 3.6. *In vivo* targeting of biomimetic NLCs

**Figure 6.**
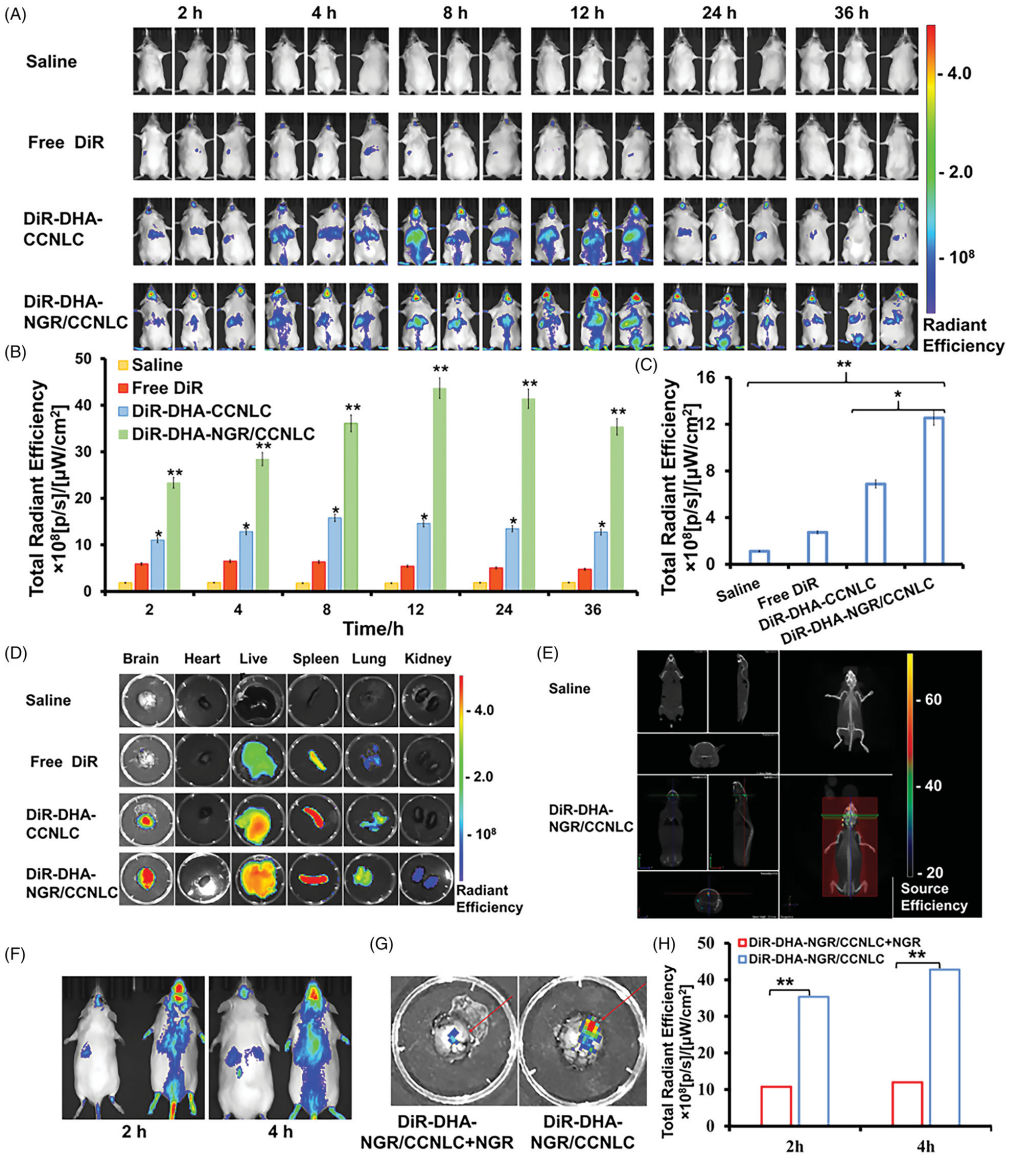
*In vivo* targeting measurement. (A) *In vivo* imaging of the saline, free DiR, DiR-DHA-CCNLC, and DiR-DHA-NGR/CCNLC groups of *in situ* glioma mice. Semi-quantitative results of the fluorescent ROI of the saline, free DiR, DiR-DHA-CCNLC, and DiR-DHA-NGR/CCNLC groups of *in situ* glioma mice (B) and (C) *in vitro* brain tissue. (D) Fluorescence imaging of isolated organs and (E) 3D brain CT localization scanning show that the biomimetic nanostructured lipid carriers can effectively target the brain. *In vivo* imaging (F) of DiR-DHA-NGR/CCNLC + NGR and DiR-DHA-NGR/CCNLC; fluorescence imaging (G) of *in vitro* brain tissue and semi-quantitative ROI results (H) (*p<.05; **p<.01).

In this study, the distribution of drugs after administration was monitored in mice in real-time via an *in vivo* imaging system. As shown in Figure 6(A,B), the fluorescence level in the saline group was weak. It is important to note that neither the fluorescence of the mice nor saline interfered with the determination of the fluorescence signals. It was difficult for the free DiR to penetrate the BBB and reach the brain, and only a small amount of fluorescence was detected in the brain, and it very quickly disappeared. DiR-DHA-CCNLC was injected into the body and circulated *in vivo*, and the fluorescence at the tumor site reached a maximum at 8 h, which was because DHA-CCNLC could be accumulated at the tumor site by homologous targeting. After 8 h, the fluorescence at the tumor site decreased gradually, but a certain fluorescence intensity was still maintained at 36 h. The DHA-NGR/CCNLC group showed obvious fluorescence at the tumor site at 2 h. The fluorescence intensity reached the highest level at 12 h and remained at high even after 36 h, which was associated with an extension of the *in vivo* circulation time by the combination of CC membrane and NGR peptide to increase the drug-brain accumulation, thus increasing the efficacy. After blocking the tumor vascular receptor with free NGR peptide, the transport of DHA-NGR/CCNLC was blocked, but it still accumulated in the tumor, proving the homologous targeting effect of CC membranes.

The *in vitro* images of various organs showed (Figure 6(C,D)) that because of the excellent BBB permeability and tumor-targeting ability of DHA-NGR/CCNLC, the fluorescence intensity at the tumor site in this group was significantly stronger than that in DHA-CCNLC group without target modification and the control group with free DiR. This proved that the biomimetic nano-preparation modified by NGR had excellent BBB-crossing and tumor-targeting abilities. Analysis of other organs found that the liver and spleen, as the largest elimination and immune organs, accumulated a large concentration of the biomimetic nanopreparations. The accumulation of drugs at the tumor site was observed by 3D CT scanning, which verified the tumor targeting ability of drugs (Figure 6(E)).

The brain targeting and tumor targeting of NGR to occupy the related receptors in the BBB in mice were verified using free NGR peptide (Vainshtein et al., 2016). As shown in Figure 6(F–H), DHA-NGR/CCNLC transport was blocked after the NGR peptide blocked the receptor, but some drugs were still transported to the brain tumor tissues via the homologous targeting mechanism. To further study BBB penetration and tumor infiltration of the biomimetic nano-preparations, the brain tissues of the mice injected with DiI-labeled biomimetic nanopreparations were sectioned. The results showed that free DiI could not penetrate the BBB, whereas DiI-DHA-CCNLC and DiI-DHA-NGR/CCNLC were distributed in tumor tissues (Figure S2A), further confirming their homologous targeting effects, with DiI-DHA-NGR/CCNLC being the most distributed at tumor sites (Figure S2B). This indicated that NGR peptide modification enhanced the tumor targeting ability of drugs.

### 3.7. *In vivo* determination of circulating time

The *in vivo* targeting experiment showed that the CC membranes could prolong the *in vivo* circulation time to a certain extent. Therefore, we measured the *in vivo* circulation time. Figure S2C shows that DiD-DHA-CCNLC and DiD-DHA-NGR/CCNLC were rapidly removed in the first 8 h, and gradually decreased after 8 h. Approximately, 20% of DiD-DHA-CCNLC and DiD-DHA-NGR/CCNLC still existed in the circulatory system at about 36 h, whereas free DiD was rapidly cleared out of the body within 4 h. The results showed that the CC membranes improved the circulation time of NLCs *in vivo*, which is beneficial in improving the bioavailability of NLCs and lays a foundation for the accumulation of NLC in the brain.

### 3.8. *In vivo* anti-glioma evaluation

The *in vivo* therapeutic performance of the biomimetic nanopreparations was studied in glioma-bearing mice. DHA-NGR/CCNLC group showed the best anti-tumor ability of all groups and significantly inhibited tumor growth compared with the saline, free DHA, DHA-NLC, and DHA-CCNLC groups.

**Figure 7.**
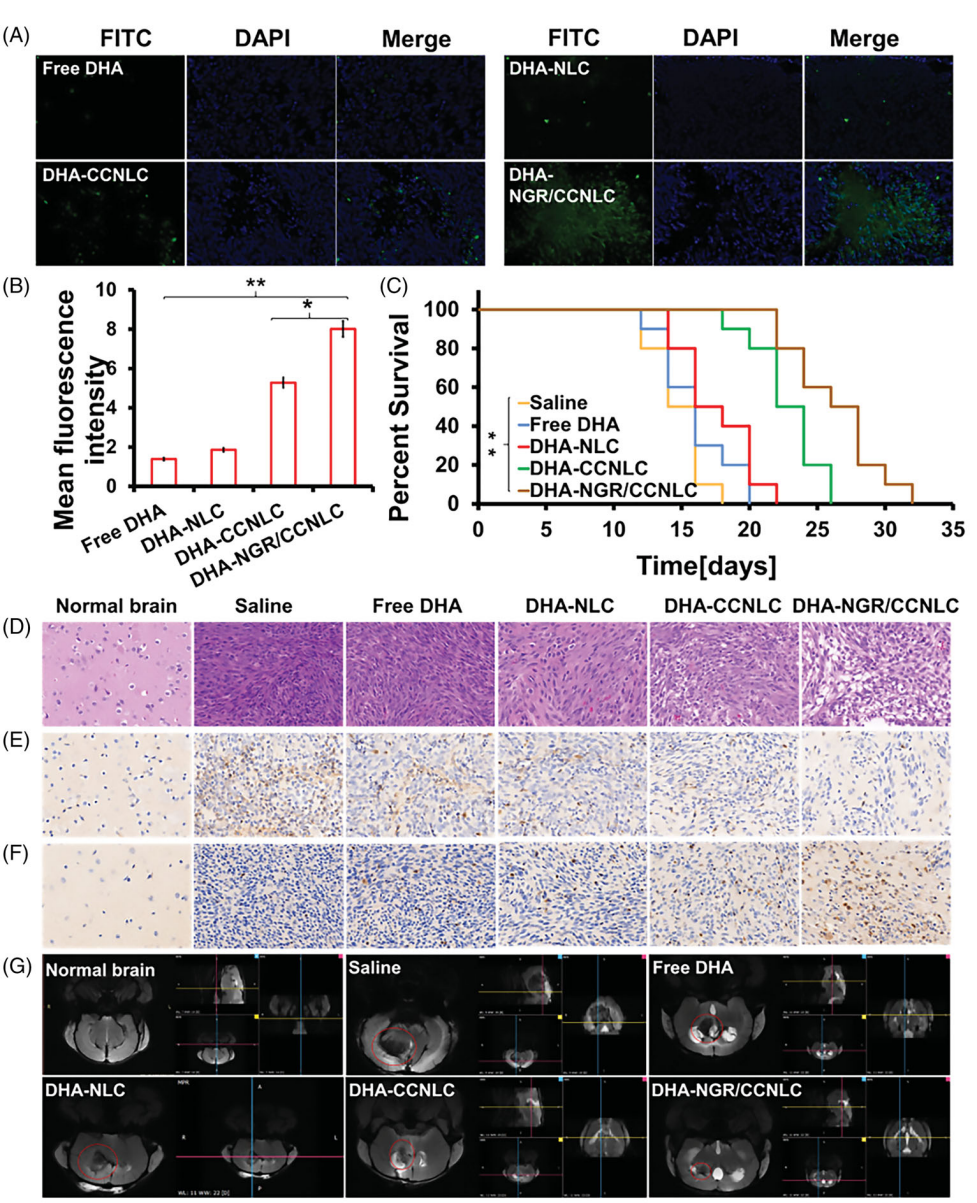
Anti-tumor effect *in vivo*. (A) TUNEL assay was used to visualize the apoptosis of the tumor tissue cells. (B) ImageJ software was used for the quantitative analysis of tumor tissue apoptosis. (C) Kaplan–Meier’s survival curves of saline, free DHA, DHA-NLC, DHA-CCNLC, and DHA-NGR/CCNLC tumor-bearing mice (*p<.05; **p<.01). (D) H&E staining of orthotopic brain tumors for histopathological analysis. (E) CD31 antibody immunofluorescence was used to determine the growth of the tumor tissue cells. (F) Caspase 3 levels were used to visualize the apoptosis of the tumor tissue cells. (G) MRI was performed to scan different sections of the brain tumors in the different administration groups. The DHA-NGR/CCNLC group had the strongest anti-tumor effect (DAPI: blue; FITC: green; CD31 and CC3: brown; ×20 objective lens).

The TUNEL results showed that (Figure 7(A)) compared with the saline and other administration groups, the DHA-NGR/CCNLC group showed significant apoptosis of the tumor cells (green), indicating that the biomimetic nanomedicine inhibited tumor growth by inducing apoptosis. ImageJ statistical quantitative results further confirmed this result (Figure 7(B)). The Kaplan–Meier survival curve also showed that DHA-NGR/CCNLC could significantly prolong the lifespan of the mice (Figure 7(C)).

Whole-brain H&E staining histological analysis showed that (Figure 7(D)) the saline group tumor cells were arranged closely, the nuclei were complete, and there was almost no damage. Further, the number of tumor cells in the free DHA and DHA-NLC groups showed some decrease, but there was no obvious damage. However, the number of tumor cells in DHA-CCNLC and DHA-NGR/CCNLC groups was significantly reduced, and significant cell shrinkage, necrosis, and tissue damage were observed. The DHA-NGR/CCNLC group had the best inhibitory effect on tumors. To investigate the proliferation and apoptosis of tumor cells, immunohistochemical staining was performed on the brain tissue sections, and the CD31 (Figure 7(E)) expression levels and analysis of apoptosis (caspase 3, CC3) (Figure 7(F)) receptor related to cell proliferation showed that the DHA-NGR/CCNLC group had the highest apoptosis and the lowest proliferation of tumor cells. On observing the development of brain tumors by nuclear magnetic resonance imaging (Figure 7(G)), DHA-CCNLC and DHA-NGR/CCNLC were found to have good anti-tumor effects. The tumor volume of mice administered with DHA-NGR/CCNLC was the smallest, and the results confirmed that the target-modified biomimetic nanomedicine had a good therapeutic effect.

### 3.9. Safety evaluation

**Figure 8.**
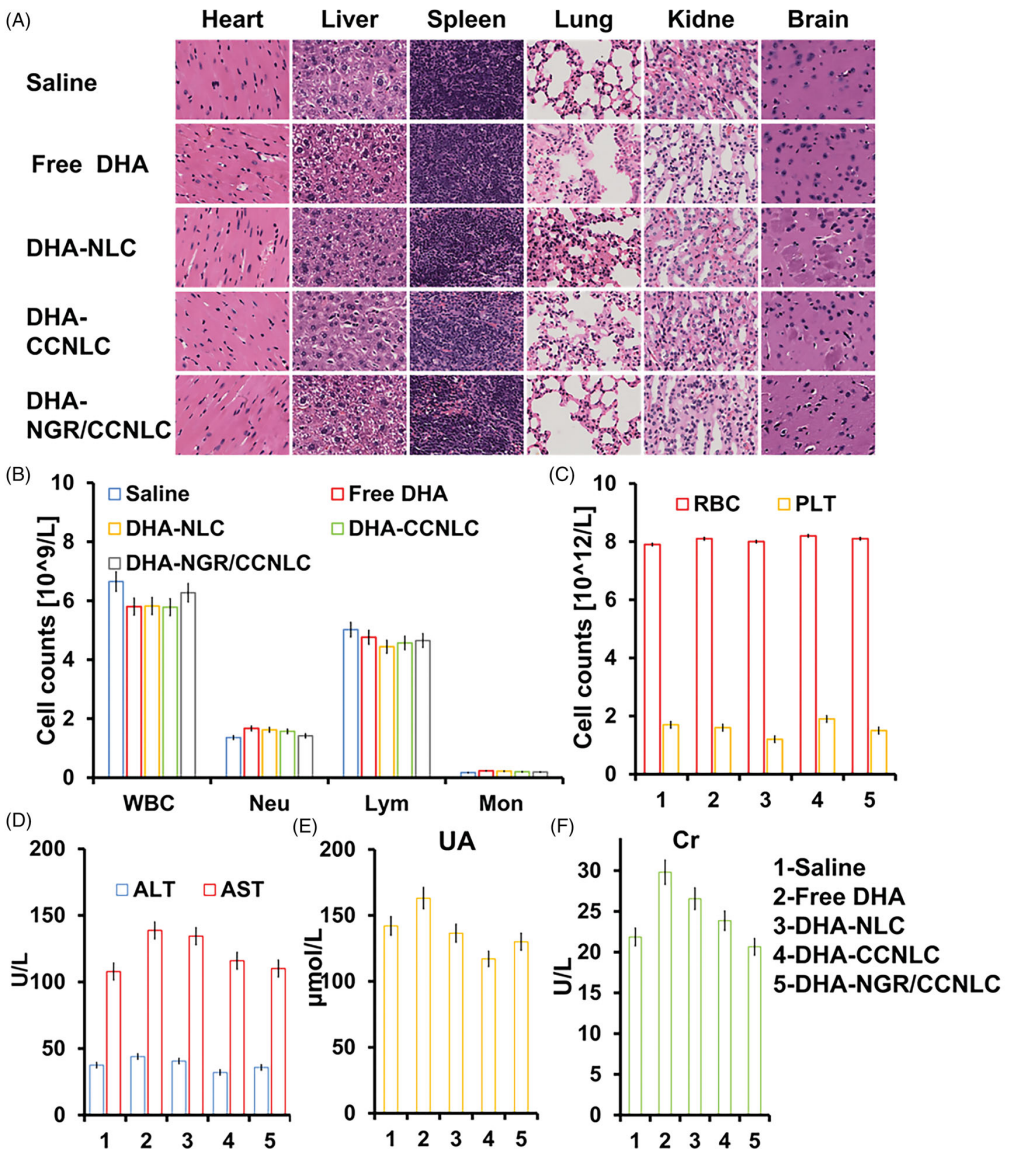
*In vivo* safety evaluation. (A) Results of H&E staining of the heart, liver, spleen, lung, kidney, and brain of mice in different administration groups were normal within the administration time. (B) Different drug administrations show no significant changes in the WBC, Neu, Lym, and Mon in mice. (C) Different administrations show little effect on the RBC and PLT in mice. Different administrations show no significant effect on serum biochemical indices including AST and ALT (D), UA (E), and Cr (F) in mice (*n* = 3, ×20 objective lens).

Histopathological analysis (Figure 8(A)) showed that there was no significant pathological damage to any organ tissues in mice injected with free DHA, DHA-NLC, DHA-CCNLC, and DHA-NGR/CCNLC compared with those injected with saline. The levels of white blood cells (WBC), neutrophils (Neu), lymphocytes (Lym), monocytes (Mon), platelets (PLT), and red blood cells (RBC) in the blood are shown in Figure 8(B,C). The changes in the indices in mice treated with each preparation were within the normal range and remained relatively stable. The levels of aspartate aminotransferase (AST), alanine aminotransferase (ALT), uric acid (UA), and creatinine (Cr) (Figure 8(D–F)) showed no significant difference between each preparation, indicating that the nano-preparations coated with CC membranes had good biocompatibility. This laid the foundation for targeted delivery to brain tumors and effective anti-tumor efficacy.

## 4. Conclusions

In the current study, we constructed a biomimetic nano-formulation encapsulated by CC membranes. DHA was prepared into DHA-NLC following a solvent volatilization ultrasonic melting method and optimized. The optimized DHA-NLC had good physical stability, a high encapsulation rate, and good controlled release performance, laying a foundation for the drug to exert its pharmacological effects. Cancer cell membranes were further used as bionic material to camouflage NLCs, and because the CC membrane itself had weak targeting ability, it was then modified with NGR peptide to improve BBB permeability and tumor targeting. The results showed that DHA-NGR/CCNLC could penetrate the BBB and BBTB, target and efficiently accumulate in the tumor tissues, and inhibit the growth of tumor cells, showing better tumor-targeting effects. In conclusion, nanosizing drugs and then modifying them using CC membranes and peptides was an effective method to enhance the anti-glioma effects of insoluble drugs. Our findings provide an effective strategy for the development of bio-nanomaterials and targeted chemotherapy for treating glioma.

## Data Availability

The [DATA TYPE] data used to support the findings of this study are available from the corresponding author upon request.
